# Intravascular leiomyomatosis with cardiac extension, a case report

**DOI:** 10.1186/s13019-023-02344-9

**Published:** 2023-09-01

**Authors:** Juan Garcés Garcés, Fernando Terán Camacho, Gerardo Dávalos Dávalos, Sofía Zárate León, Ligia Redrobán Armendáriz, Vladimir Ullauri Solórzano, Gabriel A. Molina, Santiago Endara Aguirre

**Affiliations:** 1 Radiology Service, Internal Medicine Department, Hospital Metropolitano, Quito, Ecuador; 2 Oncological Surgery, Surgery Department, Hospital Metropolitano, Quito, Ecuador; 3 Cardiothoracic Surgery, Surgery Department, Hospital Metropolitano, Quito, Ecuador; 4 General Surgery, Surgery Department, Hospital Metropolitano, Quito, Ecuador; 5 Pathology Service, Internal Medicine Department, Hospital Metropolitano, Quito, Ecuador; 6 Cardiology Service, Internal Medicine Department, Hospital Metropolitano, Quito, Ecuador; 7 General Surgery, Surgery Department, Hospital Metropolitano & USFQ, Quito, Ecuador

**Keywords:** Intravascular leiomyomatosis (IVL), Heart Tumor, Vascular growth

## Abstract

**Background:**

Intravascular leiomyomatosis (IVL) is a histologically benign smooth muscle tumor arising from the uterus that can spread through the pelvic veins and, on rare occasions, extend as far as the heart via the inferior vena cava. Despite its benign characteristics, it can behave like a malignant tumor leading to significant morbidity and even mortality if left untreated.

**Case presentation:**

The patient is a 42-year-old woman with a past medical history of uterine leiomyomas. She presented with heavy bleeding and frequent spotting; therefore, she went to her gynecologist. After further evaluation, a mass within the uterus that expanded into the pelvic veins, inferior vena cava, and right atrium was discovered. After the complete removal of the mass, the patient underwent full recovery. IVL with cardiac extension was the final diagnosis.

**Conclusion:**

Although IVL is rare, it must be considered in women who underwent previous hysterectomies or myomectomies and present with symptoms of right heart failure. The ideal therapy will need the aid of a multidisciplinary team and will depend on the patient’s symptoms, previous operative history, the tumor’s extension, and resectability.

## Introduction

Intravenous leiomyomatosis is a rare benign uterine tumor that can proliferate throughout the venous system without invading it [1]. Its treatment represents a surgical challenge since it requires the coordination of cardiothoracic, vascular, and gynecological surgeons [1, 2]. Complete excision is ideal since incomplete resection can lead to recurrence and lifelong morbidity [2, 3].

We present the case of a 42-year-old woman with a uterine mass that expanded into the pelvic veins, inferior vena cava, and right atrium. After the complete removal of the mass, the patient underwent full recovery. IVL with cardiac extension was the final diagnosis.

## Case Report

Patient is a 42-year-old woman with a past medical history of uterine leiomyomas. She underwent several fertility treatments in her youth, including ovarian stimulation, hormonal therapies (GnRH agonist), and myomectomies (two were performed at ages 30 and 33 and measured 2 cm in diameter) overall; nevertheless, all failed. She got tired of doctors and treatments so she stopped going to follow-up visits.

In recent years, her leiomyomas had grown to a worrisome size, and in the last year, she began to experience heavy bleeding, frequent spotting, and anemia, so she went to her gynecologist.

On clinical examination, an otherwise healthy patient with no signs of right-sided heart failure symptoms, including swelling and shortness of breath, was encountered; her vital signs were normal however, on her lower abdomen, a palpable mass 10 × 10 cm mass was discovered, along with some mild discomfort in the lower abdomen and pelvis. No other masses or lymph nodes were found at that time. Due to this, complementary exams were needed, Complete blood count showed hemoglobin of 11 mg/dl, EKG was normal and a contrast-enhanced computed tomography revealed a 17.6 × 7.2 × 10.3 cm uterus. It had multiple soft tissue density fibroids with central calcifications, the largest one measured 4 × 5 × 2 cm and was in close contact to the parametrial vessels.

Upon reviewing the venous phase of the CT, we were surprised to discover that within the lumen of the right internal iliac vein, the right common iliac vein, and the inferior vena cava (IVC), there was a mass causing a heterogeneous filling defect that extended from the uterus into the right atrium. All other organs were normal at that time.

**Fig. 1:**
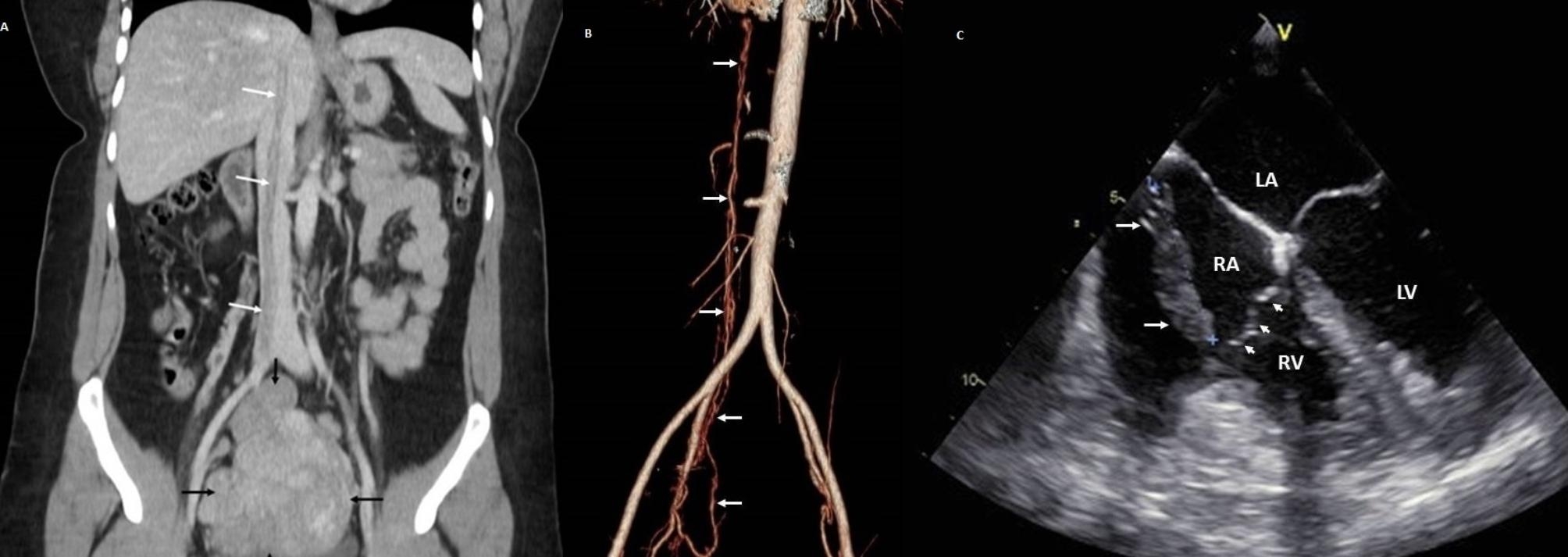
**A**: CT, filling defect on lower vena cava and mass on uterus. **B**: CT reconstruction, the whole extent of the mass is seen. **C**: Echocardiogram, the mass is seen within the right atrium

With these findings, a transesophageal echocardiogram (TEE) was done, revealing that the 3 × 1.5 cm mass moved freely into the cardiac chambers without compromising the tricuspid valve or any other structure (Fig. 1A and B). The mass reached the heart through the IVC; and moved freely with the cardiac cycle. (Fig. 1C)

The patient was admitted and was started on enoxaparin on an initial presumption of hypercoagulability. Intravascular leiomyomatosis, intravenous thrombus, Budd–Chiari syndrome, right atrial myxoma, renal cell carcinoma, and primary leiomyosarcoma were among the differential; therefore, a cardiothoracic assessment was needed. After the patient was informed of her prognosis and the possible resection of all tissues involved, a thoracic and abdominal approach was decided.

At laparotomy, a radical hysterectomy was completed, thoroughly removing the pelvic tumor; care was taken to completely remove the cardinal ligament, rectovaginal ligament, and vesicouterine ligament. Once that was completed attention was placed on the infundibulopelvic ligament, broad ligament, and then round ligament. The ureter was identified and uterine artery was cut. After amputation of the vagina, pelvic lymphadenectomy is performed.

**Fig. 2:**
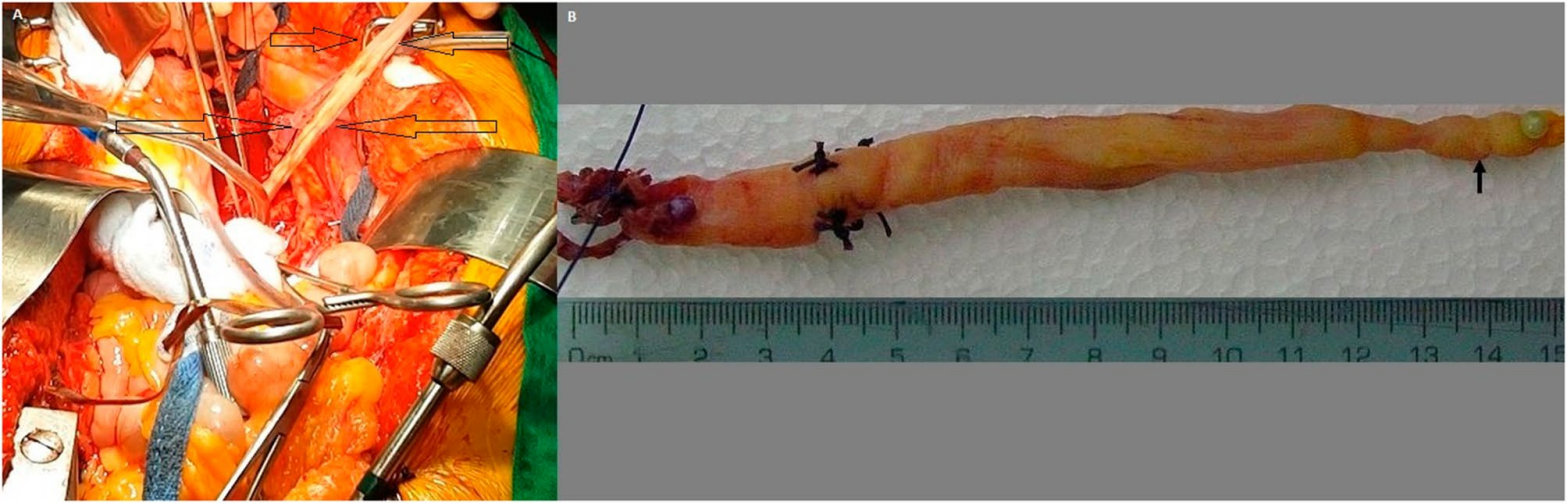
**A**: Removal of the mass from the patient, sternotomy and laparotomy is seen (arrows), **B**: The mass is completely removed from the patient’s veins

Following this, a median sternotomy and cardiopulmonary bypass (CPB) were initiated to begin the thoracic approach. Once a deep hypothermic circulatory arrest was achieved, a veno-cavotomy was done in the infrarenal IVC, which allowed the complete removal of the 5 × 1.5 cm tan-cream luminal mass from the IVC and iliac veins. The venacavatomy was closed in a one-layer fashion, and then attention was placed on the heart. The right atrium was exposed, and the 1.5 × 14 cm whiteish solid mass was removed from the right atrium and inferior vena cava under TEE guidance. Once the tumor was removed, CPB was re-established. (Fig. 2A) The heart was de-aired, and the patient was rewarmed and weaned off CPB without complications. Post CPB, another TEE examination confirmed that the cardiac chambers were free from tumors.

The excised masses consisted of 2 pieces each measuring 5 × 1.5 cm and 14 × 1.5 cm. (Fig. 2B & Supplementary Video)

**Fig. 3:**
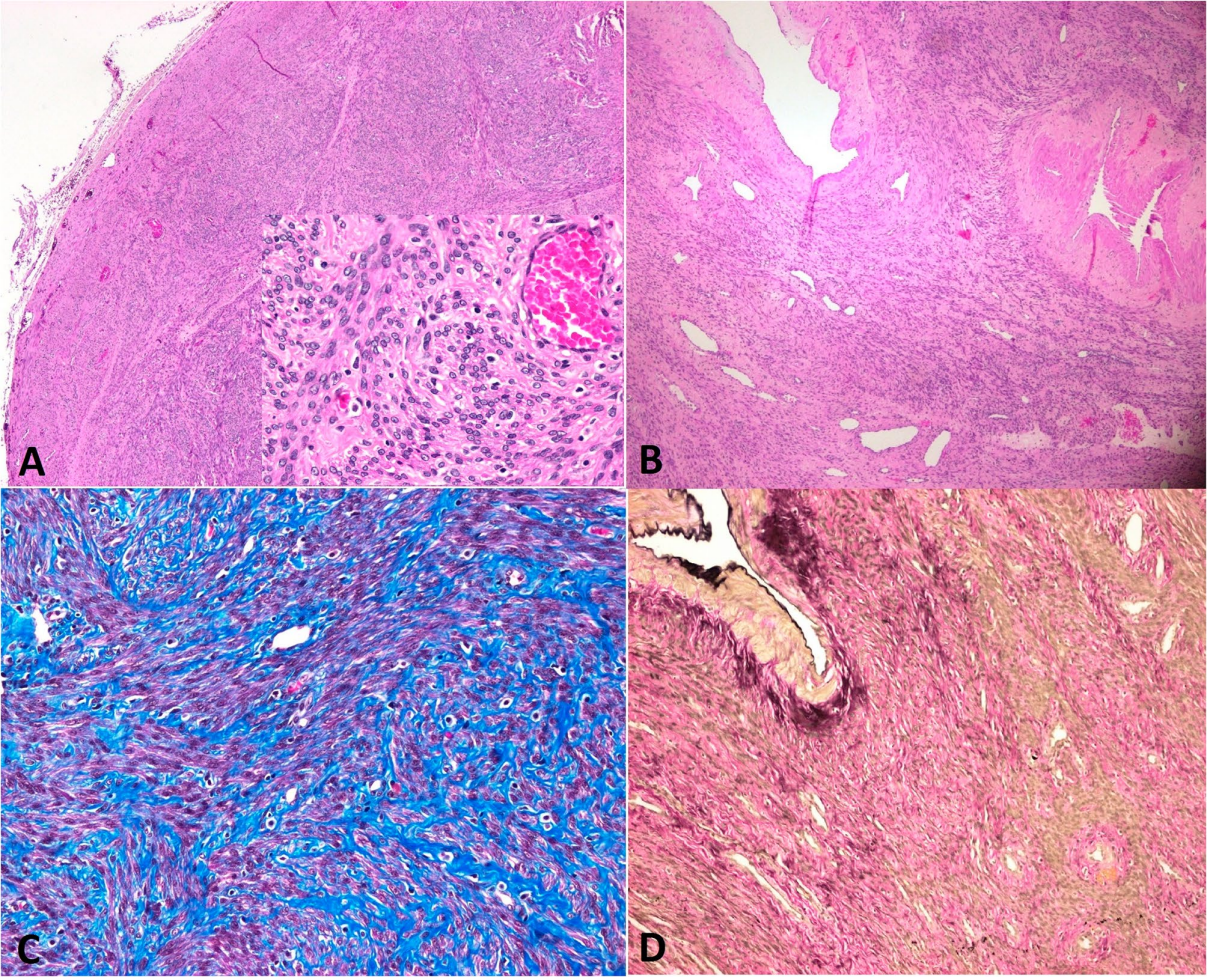
**A**: Pathology, fusocellular proliferation arranged in fascicles without atypia or mitosis is seen. **B**: Vascular structures: arteries, veins and capillary vessels seen with prominent walls. **C** and **D** Fibroid uterus showing positivity to Trichromic and Elastic 10

Pathology confirmed an intravascular leiomyoma. The masses had multiple fascicles of uniform, spindle-shaped smooth muscles without dysplasia or atypia. On immunohistochemical staining, it showed positivity for muscle actin. (Fig. 3)

The patient’s postoperative course was uneventful; she was discharged from the intensive care unit (ICU) on postoperative day three and admitted to the general ward. After she tolerated a full diet, she was discharged under strict surveillance and with long-term nonsteroidal estrogen receptor antagonist therapy.

Two years after the initial surgery, the patient is doing well without signs of recurrence.

## Discussion

Intravascular leiomyomatosis (IVL) is an extremely rare mesodermal cell tumor [1]. Since the first description of IVL in 1896, fewer than 300 cases have been reported in the English literature [1, 2]. It primarily affects women of reproductive age (30%) and has extrauterine involvement in about 30% of cases [1, 3]. As it was found in our patient.

This disease can grow over many years from the uterus into the pelvic vein, renal vein, vena cava, and finally into the right atrium [2, 3]. In a few reported cases, the tumor can even travel into the right ventricle and pulmonary artery through the tricuspid valve, resulting in sudden death [1]. Nonetheless, intracardiac extension is extremely rare and, since first described by Dürck in 1907, accounts for only about 10% of all cases [4]. The pathogenesis of IVL is not yet fully understood [1, 4]. However, it is thought that the origin of IVL happens because there is an abnormal abdominal vascular invasion of the myometrial leiomyoma into the walls of the veins [1, 2]. Further investigations from Quade et al. and Dal Cin et al. have suggested chromosomal aberrations that may lead to intravascular intrusion and proliferation [5]. It can also appear due to a hormonal stimulus, as some patients with IVL underwent fertility treatments and had twin pregnancies [1, 5]. As it happened to our patient.

The clinical manifestations of IVL are related to the extent of involvement [1, 3]. When the tumor is confined to the uterus, patients may be asymptomatic or experience abdominal pain, bleeding, leucorrhea, and abdominal mass [4]. Lower limb edema, ascites, thrombosis, and hepatosplenomegaly may occur as the disease progresses and affects the inferior vena cava; when the tumor reaches the right atrium, it can cause palpitations, dyspnea, and syncope [1, 3]. Since the symptoms are broad and non-specific, a delay in diagnosis can occur; therefore, a thorough understanding of this pathology is needed to reach the final diagnosis [4, 6]. In our case, she had bleeding and anemia. Nonetheless, no history of right-sided heart failure was found at the time.

Macroscopically, intravenous leiomyomatosis is a nodular growth with wormlike extensions into the uterine veins [1, 7]. Histopathologically, it shows benign smooth muscle growing within the veins [1, 8]. It can be classified into many variants, such as cellular, atypical, epithelioid, myxoid, and lipoleiomyoma [1, 3, 5].

Ultrasound, doppler, CT, and Magnetic resonance imaging are needed for diagnosis and confirm disease extension [5, 6]. These tumors are usually seen as an elongated mobile mass extending from the veins of the lower body into the right heart chambers [1, 5, 7]. In our patient, during the CT, we discovered the mass within the patient’s veins that completely changed our approach and our initial diagnosis.

Surgery is the most effective treatment, but it depends on whether the tumor can be excised entirely [2, 4]. Since 1982, two surgical procedures have been performed to treat intravascular leiomyomatosis with heart extension [9, 10]. The first requires completely removing the intracardiac and intracaval mass through a one-stage surgical approach, and the other requires a two-stage abdominal stage and a thoracic stage; the approach will depend on the preoperative status of the patient and the extension of the tumor [1, 2]. Yet, the two-stage surgery is usually preferred because of its shorter operation time, reduced risk of bleeding, and safer resection of intracardiac tumor masses [6, 7]. As it was done in our patient.

Complete removal is the only way to ensure a favorable prognosis, yet, close surveillance is needed as tumor recurrence (22.2 to 30%) can occur up to 15 years after the surgery. (10, 11) Postoperative hormonal therapy with antiestrogens, aromatase inhibitors, hysterectomy, or bilateral salpingo-oophorectomy is recommended to prevent this complication. (1, 11) As it was done with our patient.

IVL is a rare, aggressive condition that, due to its indolent course, can range from being entirely asymptomatic to experiencing symptoms of right heart failure. Therefore, early detection and close surveillance are vital to ensure a good recovery.

## Conclusion

Although IVL is rare, it needs to be in our differential. Especially on women who underwent previous hysterectomies or myomectomies. The ideal treatment will require the joint efforts of a multidisciplinary team and will depend on the patient’s symptoms, previous operative history, the tumor’s extension, and resectability. If complete resection is achieved, recurrence is rare, yet if complete removal is not achieved, recurrence can appear in up to a quarter of patients.

## Data Availability

The data is available to the editor, the images are our own.

## References

[1] Xu Y, Gao X, Yang C, Liu J, Jin B, Shang D (2020). Intravascular leiomyomatosis extending to Right Atrium: a rare caused Syncope. Ann Vasc Surg.

[2] Chen YL, Zheng A, Han L (2022). Intravascular leiomyomatosis with intracardiac extension: a case report. Asian J Surg.

[3] Zaidi AZ, Hawley I, Zaidi J (2021). Intravenous leiomyomatosis-a case report. J Obstet gynaecology: J Inst Obstet Gynecol.

[4] Ghanem M, Meyer F, Jechorek D, Schoeder V, Ignatov A, Fadel M, Halloul Z (2019). Intravascular (post-hysterectomy) leiomyoma (IVL) as late tumor thrombus within the inferior vena cava (IVC)-A rare case primarily imposing as IVC thrombus originating from left renal vein after former left nephrectomy status. Pathol Res Pract.

[5] Ordulu Z, Nucci MR, Cin D, Hollowell P, Otis ML, Hornick CN, Park JL, Kim PJ, Quade TM, B. J., Morton CC, Li R, Shen Y, Sun Y, Zhang C, Yang Y, Yang J, Su R, Jiang B. (2014). Intravenous leiomyomatosis with intracardiac extension: echocardiographic study and literature review. Texas Heart Institute journal, 41(5), 502–506. https://doi.org/10.14503/THIJ-13-3533. 10.14503/THIJ-13-3533 PMC4189351 25425982

[6] Deng Y, Dong S, Song B (2021). Surgical Strategy for Intravenous Cardiac Leiomyomatosis. Heart Lung Circ.

[7] Liu N, Long Y, Liu Y (2020). Intravenous leiomyomatosis: Case series and review of the literature. J Int Med Res.

[8] Bahary CM, Gorodeski IG, Nilly M, Neri A, Avidor I, Garti IJ (1982). Intravascular leiomyomatosis. Obstet Gynecol.

[9] Stegmann T, Garcia-Gallont R, Döring W (1987). Intravascular leiomyomatosis: report of a case and review of the literature. Thorac Cardiovasc Surg.

[10] Declas E, Lucot JP (2019). La léiomyomatose extra-utérine: revue de la littérature. Gynecologie obstetrique fertilite & senologie.

